# Single-Neuron Labeling in Fixed Tissue and Targeted Volume Electron Microscopy

**DOI:** 10.3389/fnana.2022.852057

**Published:** 2022-04-21

**Authors:** Marta Turegano-Lopez, Andrea Santuy, Asta Kastanauskaite, Jose-Rodrigo Rodriguez, Javier DeFelipe, Angel Merchan-Perez

**Affiliations:** ^1^Laboratorio Cajal de Circuitos Corticales, Centro de Tecnología Biomédica, Universidad Politécnica de Madrid, Pozuelo de Alarcón, Spain; ^2^Ph.D. Program in Neuroscience, Universidad Autónoma de Madrid – Instituto Cajal, Madrid, Spain; ^3^Department of Neurology and Epileptology, Hertie Institute for Clinical Brain Research, University of Tübingen, Tübingen, Germany; ^4^Instituto Cajal, Consejo Superior de Investigaciones Científicas, Madrid, Spain; ^5^Centro de Investigación Biomédica en Red sobre Enfermedades Neurodegenerativas (CIBERNED), ISCIII, Madrid, Spain; ^6^Departamento de Arquitectura y Tecnología de Sistemas Informáticos, Universidad Politécnica de Madrid, Madrid, Spain

**Keywords:** FIB-SEM, serial sectioning, 3D reconstruction, intracellular injection, quantitative neuroanatomy

## Abstract

The structural complexity of nervous tissue makes it very difficult to unravel the connectivity between neural elements at different scales. Numerous methods are available to trace long-range projections at the light microscopic level, and to identify the actual synaptic connections at the electron microscopic level. However, correlating mesoscopic and nanoscopic scales in the same cell, cell population or brain region is a problematic, laborious and technically demanding task. Here we present an effective method for the 3D reconstruction of labeled subcellular structures at the ultrastructural level, after single-neuron labeling in fixed tissue. The brain is fixed by intracardial perfusion of aldehydes and thick vibratome sections (250 μm) are obtained. Single cells in these vibratome sections are intracellularly injected with horseradish peroxidase (HRP), so that the cell body and its processes can be identified. The thick sections are later flat-embedded in epoxy resin and re-sectioned into a series of thinner (7 μm) sections. The sections containing the regions of interest of the labeled cells are then imaged with automated focused ion beam milling and scanning electron microscopy (FIB-SEM), acquiring long series of high-resolution images that can be reconstructed, visualized, and analyzed in 3D. With this methodology, we can accurately select any cellular segment at the light microscopic level (e.g., proximal, intermediate or distal dendrites, collateral branches, axonal segments, etc.) and analyze its synaptic connections at the electron microscopic level, along with other ultrastructural features. Thus, this method not only facilitates the mapping of the synaptic connectivity of single-labeled neurons, but also the analysis of the surrounding neuropil. Since the labeled processes can be located at different layers or subregions, this method can also be used to obtain data on the differences in local synaptic organization that may exist at different portions of the labeled neurons.

## Introduction

The relatively high density and complex arrangement of cells that make up the nervous system, their extensive dendritic and axonal arborizations and the complexity of neuronal connections, all make it extremely difficult, if not impossible, to study completely —and in detail— both the microscopic and ultrastructural characteristics of any cell of the nervous system. The introduction of the combined Golgi-electron microscope technique in the 1970s and 1980s represented a major stimulus to search for methods that permit cells identified at the light microscopic level to be studied subsequently by electron microscopy. This allowed the first steps to be taken toward the reconstruction of complex neuronal circuits (reviewed in Blackstad, 1970, 1981; Freund and Somogyi, 1989; Fairén, 2005).

Since then, important advances have been made in the study of the neural circuits thanks to the development of a variety of light and electron microscope techniques, including immunocytochemistry; combinations of tract-tracing methods and histochemistry or immunocytochemistry; intracellular injections of dyes; and genetic methods to trace brain circuits. The reconstruction of subcellular structures in 3D usually requires serial sectioning and obtaining such series of sections manually is both difficult and time-consuming. To overcome these limitations, automated electron microscopy methods have been developed that allow the acquisition of long series of sections. Although transmission electron microscopy techniques have been developed (Yin et al., 2020), most of these automated methods are based on scanning electron microscopy (SEM) (Kornfeld and Denk, 2018). They include serial block-face SEM (Denk and Horstmann, 2004; Briggman and Denk, 2006), array tomography (Micheva and Smith, 2007), automated tape-collecting ultramicrotomy (ATUM) (Schalek et al., 2011; Hayworth et al., 2014), focused ion beam milling SEM (FIB-SEM) (Knott et al., 2008; Merchán-Pérez et al., 2009), and cluster ion beam SEM (Hayworth et al., 2020).

The main challenge is to correlate the findings obtained by the methods that operate at a scale of hundreds or thousands of microns and those operating at a scale of nanometers. Ideally, a given cell can be identified at the light microscopic level on the basis of its location, cytoarchitecture, long-range connectivity or other criteria, and then studied at the ultrastructural level to obtain further information, especially regarding its connectivity at the synaptic level. Bridging the gap between light and electron microscopy is, however, a laborious and technically demanding task, since it requires compatible processing methodologies for both scales and a high degree of expertise in the different techniques needed. Moreover, it is important that the approach is accurate enough to ensure that the same location that is observed at the light microscopic field is later observed at the electron microscopic level (e.g., DeFelipe and Fairén, 1982).

**FIGURE 1:**
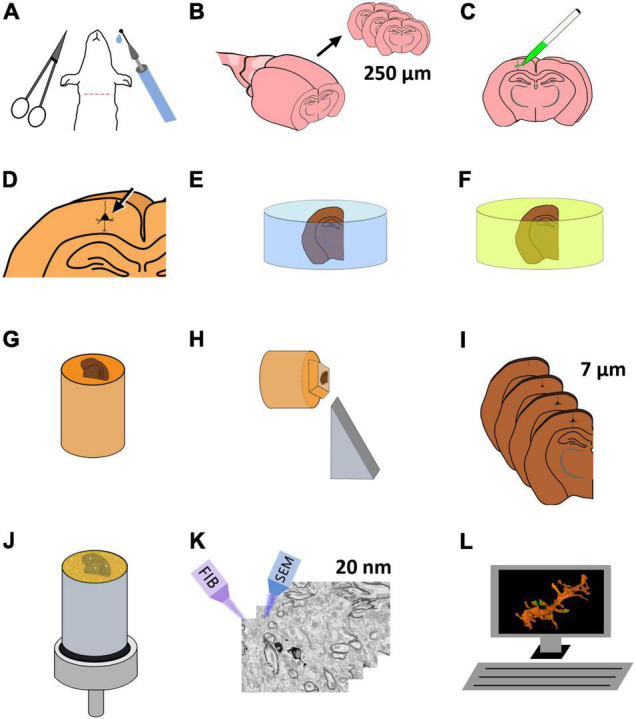
Schematic representation of the protocol for the preparation of brain tissue for single-neuron labeling and FIB-SEM imaging. **(A)** Perfusion fixation of experimental animals. **(B)** Vibratome sectioning in slices (250 μm thick). **(C)** Intracellular injection of HRP and LY. **(D)** Development of HRP. **(E)** Postfixation and osmication. **(F)** En bloc staining. **(G)** The flat embedded section is glued onto a blank Araldite block. **(H)** Re-sectioning of the sample. **(I)** A series of 7 μm sections is obtained. **(J)** One of the 7 μm sections is selected and mounted for FIB-SEM. To facilitate charge dissipation, the block (except for the upper surface) is painted with conductive silver paint, and then sputter-covered with gold-palladium. **(K)** FIB-SEM serial milling and imaging. **(L)** Three-dimensional reconstruction and image analysis.

In the present article, we present a method to correlate light and electron microscopy by labeling identified single cells in fixed sections of brain tissue. Selected portions of the labeled cells can later be imaged at the electron microscopic level and reconstructed in three dimensions. Single-cell labeling is performed by intracellular injection of horseradish peroxidase (HRP) in fixed vibratome sections (250 μm thick). The injections are performed under a fluorescence microscope, and the selection of the cells to be injected is facilitated by nuclear staining. The injection is monitored by injecting a fluorescent tracer together with the HRP. After development of the injected HRP, the vibratome section is flat-embedded in an epoxy resin. The vibratome section is then re-sectioned into a series of thinner, 7 μm sections, so that the region of interest can be accurately located. The specific section containing the region of interest is further sectioned and imaged, this time at the ultrastructural level, using FIB-SEM, obtaining a series of EM images that can later be reconstructed in 3D and analyzed (Figure 1).

### Equipment

The main equipment used to set up the technique was as follows: Vibratome (Leica VT 1200S); Electrode Puller (Sutter Instrument Co., Model P-97) and glass capillary electrodes (1 mm × 58 mm, A-M Systems, catalog #601000); Fluorescence Microscope (Olympus BX51WI) equipped with a Green Fluorescent Protein (GFP) filter set and Leica micromanipulators; Microiontophoresis Current Generator (World Precision Instruments, model 260); Rotating Automated Microtome (Thermo Scientific, Microm HM 360); Tungsten Carbide Knives (Ted Pella #121-50); Sputter Coater (Quorum Emitech SC7620); and Focused Ion Beam – Scanning Electron Microscope (FIB-SEM; Zeiss, CrossBeam 540).

### Solutions

Perfusion fixation solution: prepare 4% paraformaldehyde (PFA; Aldrich #441244) and 0.125% glutaraldehyde (GA; TAAB #G002) in 0.1 M phosphate buffer pH 7.4 (PB). The solution must be prepared just before use in a fume hood.

First postfixation solution: 0.125% GA in PB. Prepare just before use in a fume hood.

Nuclear staining solution: dissolve 1 μl of SYTOX Green (Thermo Fisher #S7020) in 1 ml of phosphate buffered saline (PBS), pH 7.4. Store at 4°C.

Stock solution of fluorescent tracer: 8% Lucifer Yellow (LY; Sigma #L0259) in 0.1 M TRIS buffer, pH 7.4. Store at 4°C.

Stock solution of HRP: prepare an 8% solution of HRP by dissolving 5 mg of HRP (Sigma-Aldrich P8375-5KU) in 63 μl of 0.05 M TRIS buffer, pH 7.4. Prepare 5 μl aliquots and store at −20°C.

Intracellular injection solution: mix 1 μl of the stock solution of HRP and 2 μl of the stock solution of LY (the final concentration of HRP is 2.67%). Mix well and centrifuge for 5 min before electrode filling.

Preincubation solution of 3,3′-Diaminobenzidine (DAB): the solution contains 10 mg of DAB (Sigma #D5905) and 200 μl of dimethyl sulfoxide (DMSO, Sigma D8418) in 20 ml of PB. Prepare immediately before use in a fume hood and protect from light. Filter with a syringe filter (Acrodisc 0.2 μm, #4612) before use.

Incubation solution of DAB: immediately before use, add 5 μl of 30% H_2_O_2_ (Merck # 1.07209.1000) to 10 ml of the DAB solution described above and mix well.

Second postfixation solution: freshly prepared 2% PFA, 2.5% GA, and 3 mM CaCl_2_ (Sigma-Aldrich #C2661) in 0.1 M cacodylate buffer. Prepare in a fume hood.

Osmium solution: 1% osmium tetroxide (Sigma-Aldrich #O5500), 7% glucose (Merck #1.08337.0250) and 3 mM CaCl_2_ in 0.1 M cacodylate buffer. Always handle osmium in a fume hood, with protective glasses and gloves.

Uranyl acetate solution for *en bloc* staining: dissolve 1% uranyl acetate (Electron Microscopy Sciences #22400) in 50% ethanol. Filter with a syringe filter (Acrodisc 0.2 μm, #4612).

## Methods

### Animals, Perfusion Fixation, and Vibratome Sectioning

Four adult male mice (C57BL/6, 8 weeks old) were used to set up the technique presented here. All animals were handled in accordance with the guidelines for animal research set out in the European Community Directive 2010/63/EU, and all procedures were approved by the Local Ethics Committee of the Spanish National Research Council (CSIC). The animals were deeply anesthetized with an intraperitoneal injection of pentobarbital (40 mg/kg) and intracardially perfused with 200 mL of freshly prepared fixation solution (4% PFA, 0.125% GA, in PB). The brains were postfixed for 6–16 h (overnight) in the first postfixation solution (0.125% GA). Vibratome sections (250 μm thick) were obtained and collected in PB in 24-well flat-bottom plates.

### Intracellular Injection of Horseradish Peroxidase and Lucifer Yellow

In order to visualize the cell nuclei, the vibratome section to be injected is placed in SYTOX Green solution (SG) for 30 s, and then placed in a methacrylate dish in PBS until injection. The intracellular injection solution is prepared by mixing the stock solutions of LY and HRP, as described above. The tip of the microelectrode is filled with the HRP-LY mix, and a conductive solution of 0.1 M lithium chloride is used to fill the rest of the microelectrode. The sections are observed under a fluorescence microscope equipped with a GFP filter set. The region of interest and the cells to be injected are selected making use of the nuclear staining with SG. Cell nuclei are used as a guide for the impalement of the cell body with the microelectrode. When current injection is turned on, LY allows visual monitoring of the quality of impalement and of the progress of cell filling. Once we have visually ascertained that LY is correctly filling the cell, the current polarity is reversed in cycles of 30 s to 4 min. The filling process is periodically monitored until the cell is completely filled. An arbitrary number of cells can be injected in each vibratome slice. We recommend, however, keeping enough distance between them to avoid overlapping of cell arborizations. After the desired cells have been injected, the vibratome section is washed three times in PBS, for 5 min each time, and stored at 4°C in the dark until development of HRP.

### Horseradish Peroxidase Development

For HRP development, wash three times in PB at room temperature, 5 min each time. Preincubate the vibratome section in a plastic Petri dish for 15 min in DAB solution without H_2_O_2_, in a fume hood with mild agitation, protected from light. Next, incubate in DAB solution with H_2_O_2_ for 5–10 min. Progress of DAB precipitation can be periodically monitored under an optical microscope. When the precipitate has reached the desired intensity, the reaction is stopped by washing the sections three times in PB, 5 min each time.

### Postfixation and Osmication

Wash the vibratome section (three times, 10 min each) in 0.1 M cacodylate buffer, pH 7.4. The section is postfixed for 2 h in the second postfixation solution (2% PFA, 2.5% GA, and 3 mM CaCl2 in 0.1 M cacodylate buffer). Add the fixative carefully with a plastic Pasteur pipette and do not agitate to prevent sections from curling or folding. Handle in a fume hood. Wash three times, 10 min each time, in 0.1 M cacodylate buffer. Osmicate for 1 h in the osmium solution, always in a fume hood, with protective glasses and gloves. Add and remove the osmium solution very slowly with a plastic Pasteur pipette to prevent sections from folding or breaking. During osmication, the sections become brittle and break easily, so use a small spatula or weighing spoon to handle them carefully. Wash three times in cacodylate buffer, 10 min each time, and store overnight in the refrigerator.

### *En bloc* Staining, Flat Embedding, and Re-sectioning

**FIGURE 2:**
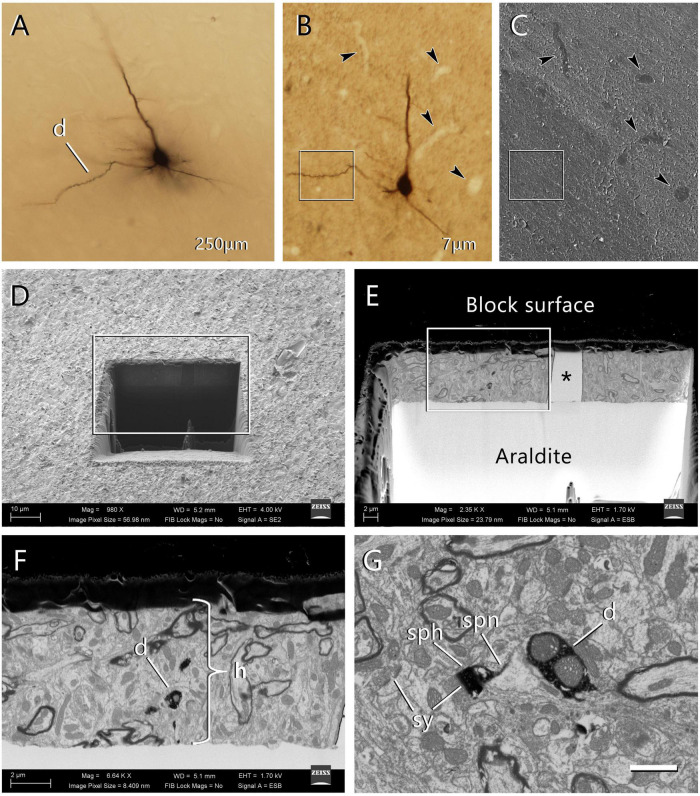
Selection of the region of interest in a labeled cell and FIB-SEM imaging. **(A)** An intracellularly injected pyramidal neuron in a 250 μm-thick vibratome section. The labeled cell was injected with HRP, developed with DAB and flat-embedded in Araldite. One dendritic segment (d) was selected for further study. In this particular example, the injected cell was a pyramidal neuron from layer three of the motor cortex. **(B)** One of the 7 μm-thick serial sections that were obtained from the 250 μm-thick vibratome section. This section contains the selected dendritic segment (inset). Blood vessels (arrowheads) were clearly identifiable and were later used as landmarks. Once this section was photographed, it was mounted on a blank Araldite block and observed with the SEM. **(C)** The surface of the 7 μm section photographed with the SEM using the secondary electron detector. The same blood vessels were visible (arrowheads), so they were used as landmarks to identify the region where the labeled dendritic segment is located (inset). **(D)** A viewing trench was excavated with the FIB in the 7 μm-thick section to gain visual access to the target dendrite. This image was taken with the secondary electron detector. The inset indicates the region that is further magnified in the next panel. **(E)** Backscattered electron image showing the trench that was excavated with the FIB to identify the selected dendritic segment. The gray scale has been inverted so the block surface appears dark and the Araldite bed appears light. The visible portion of brain tissue is crossed vertically by a blood vessel (asterisk). The region within the inset is further magnified in the next panel. **(F)** Region of interest where the labeled dendrite (d) is visible due to the dark, electron-dense precipitate. The thickness of the section (h) is approximately 7 μm. **(G)** Detail of one of the serial images acquired from the dendrite (d). One of its dendritic spines is visible, showing both the spine head (sph) and spine neck (spn). Synaptic junctions (sy) can be identified on the dendritic spine and in the surrounding tissue. Calibration bar in **(G)** 50 μm for **(A–C)**; 16 μm for **(D)**; 6.9 μm for **(E)**; 2.4 μm for **(F)**; and 1 μm for **(G)**.

*En bloc* staining is performed by placing the sections in the uranyl acetate solution at 37°C for 30 min. Dehydrate in a graded series of ethanol, clear in acetone and flat-embed in Araldite. Any other resin that is suitable for electron microscopy (Durcupan, Spurr, etc.) may also be used. Once the resin has cured, the flat-embedded section is examined and photographed under an optical microscope (Figure 2A). The region of interest is then trimmed and glued with cyanoacrylate onto a blank Araldite block. We then use a tungsten carbide knife (Ted Pella #121-50) to obtain serial sections (7 μm thick), collecting them on glass slides that have been previously covered with silicone and Araldite. Once the series of 7 μm sections have been obtained, they are examined and photographed to select the region of interest. Blood vessels and other morphological features in and around the region of interest will later be used as landmarks to precisely locate the area to be imaged with the FIB-SEM (Figure 2B).

### Focused Ion Beam Milling and Scanning Electron Microscopy Imaging

Once the region of interest has been selected in the 7 μm section, the section is detached from the glass slide and re-mounted on a blank Araldite block with cyanoacrylate glue. The new block is mounted on an SEM specimen stub with a conductive carbon sticker (Electron Microscopy Sciences, #77825-09). To prevent charge build-up, the block is covered with silver paint (Electron Microscopy Sciences, #12630), except for the top surface. It is important not to cover or spill silver droplets on the upper surface of the block where the specimen is located. Conversely, the base of the block must be carefully painted to ensure electrical continuity between the Araldite block and the specimen stub. Let the paint dry for at least 24 h in a vacuum desiccator. Charge dissipation from the upper surface of the block is achieved by gold-palladium sputter-coating for 30 s. Carbon, gold alone, or other metals are also suitable for sputter coating, but care must be taken not to cover the specimen with a layer that is too thick as this might obscure surface details.

The surface of the block is then photographed with the SEM using the secondary electron detector. The landmarks in the section that were previously identified with the optical microscope (mainly small blood vessels) are also visible with the SEM, so the region of interest can be precisely located (Figure 2C). A viewing trench is then excavated with the FIB using a 7 nA milling current, to provide visual access to the region that we plan to image. The front face of this trench must be located close enough to the target to allow its identification (Figures 2D–F). The ion beam and the electron beam can be used simultaneously, so it is possible to monitor the progression of the trench as it is being excavated. In practice, however, it is difficult to find the labeled structure while continuing the excavation, so we usually pause the milling process periodically. During these pauses, we examine the front face of the trench in search of the labeled structure, and we acquire an image using the back-scattered electron detector (0.6–2.0 kV acceleration voltage). As soon as we have identified our target, milling of the viewing trench is stopped. We then use a smaller FIB current (700 pA) to progressively mill the front face of the trench in steps of 20 nm. During each milling step, we remove 20 nm of material with the FIB, and then use the SEM to take a microphotograph of the freshly milled surface (Figure 2G). In our equipment, the angle between the SEM and the FIB is 54°, so the angle of incidence of the SEM on the surface to be imaged is 54°, rather than perpendicular. The resulting perspective deformation is automatically corrected by the microscope software during acquisition (SmartSEM 6.02; Carl Zeiss Microscopy Ltd.), so no distortion is present in the final images.

**FIGURE 3:**
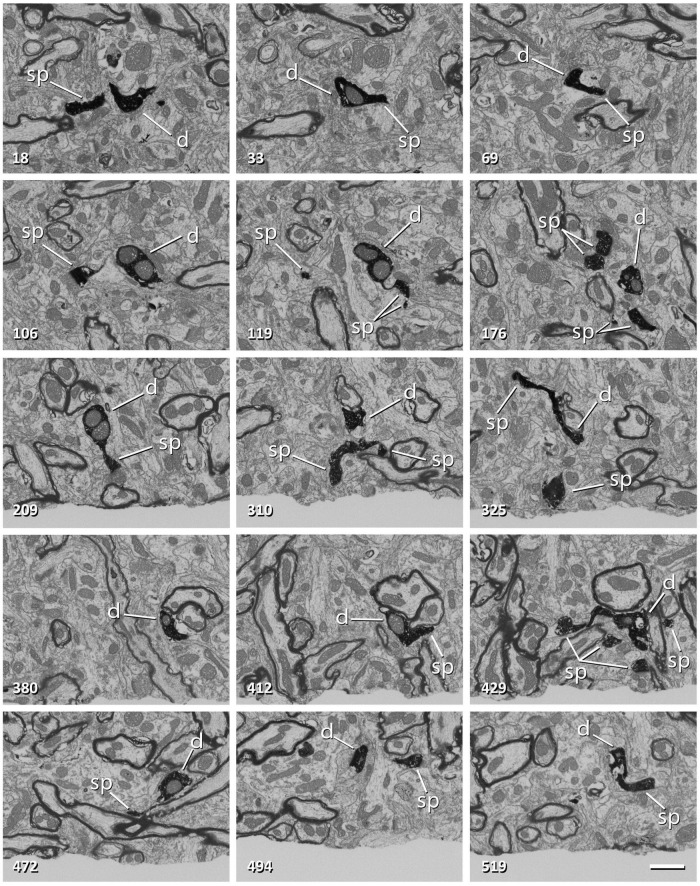
Non-consecutive images of a labeled dendrite from a series obtained with FIB-SEM. The number in the bottom-left corner indicates the position of each image in the series. The dendritic shaft (d) and dendritic spines (sp) are filled with a dark electron-dense precipitate. Note that frame 106 corresponds to Figure 2G. Scale bar, 1 μm. See also Supplementary Video 1.

Since the milling/imaging cycle can be fully automated, serial images of the target are obtained (Figure 3). We routinely use a milling step of 20 nm (equivalent to section thickness) and a resolution in the *X*-*Y* plane of 5 nm/pixel, so the actual voxel size is 5 nm × 5 nm × 20 nm. Other resolutions and milling steps can also be used, depending on the particular imaging needs, and the length of the series of sections can be selected according to the researcher’s needs. During the development of the technique, we performed five series of FIB-SEM sections at different locations to be certain that any selected cell region can be located, accessed and imaged.

## Results

Long series of consecutive EM images can be obtained from any region of interest that has been previously selected at the light microscopic level. In the particular example presented here, a relatively long dendritic segment was selected for study, obtaining a series of 569 images (Figures 2F,G, 3 and Supplementary Videos 1, 2). Given that the size of each image was 2,048 × 1,536 pixels and was acquired with a resolution of 5 nm/pixel, the field of view was 10.24 μm × 7.68 μm. Since the spacing between consecutive sections was 20 nm, the size of the stack was 10.24 μm × 7.68 μm × 11.38 μm, with sufficient resolution to identify small subcellular structures such as synaptic vesicles.

Since some drift takes place during acquisition of the FIB-SEM image series, further alignment (registration) is necessary. For the registration, we used FIJI, a distribution of ImageJ with preinstalled plugins for microscopy (Schindelin et al., 2012)^1^. We routinely use a “rigid” registration protocol to avoid deformation of individual images. Also, alignment is performed by translation only, with no rotation allowed. The aligned stack of images is then loaded into Espina software (Morales et al., 2011), which allows visualization through the original plane of section or the other two orthogonal planes (Espina software can be downloaded from https://cajalbbp.es/espina/).

The DAB precipitate appears as a dark, granular and very electron-dense material that fills the dendritic shaft and the dendritic spines (Figure 3 and Supplementary Videos 1, 2). The labeled dendrite is thus easily recognizable, and can be followed throughout the stack of serial sections. The dark intracellular deposit fills most of the cytosolic space, masking small organelles, although other structures such as mitochondria, multivesicular bodies or large vacuoles are clearly identifiable. The surrounding tissue is lighter, except for myelin sheaths, which form a dark, smooth covering around some axons. Synapses are identified in serial sections by the presence of synaptic vesicles in the presynaptic axon and electron-dense pre- and postsynaptic densities (Merchán-Pérez et al., 2009). The fact that the postsynaptic densities appear in several consecutive images greatly facilitates their identification. Also, the postsynaptic densities are membrane-bound and smooth, while the DAB precipitate is intracellular and granular. Synaptic junctions with a prominent postsynaptic density are classified as “asymmetric” and synapses with a thin postsynaptic density as “symmetric” (Gray, 1959; Colonnier, 1968). In the cerebral cortex, asymmetric and symmetric synapses correspond to excitatory (glutamatergic) and inhibitory (GABAergic) synapses, respectively (Houser et al., 1984; Peters et al., 1991; Ascoli et al., 2008).

**FIGURE 4:**
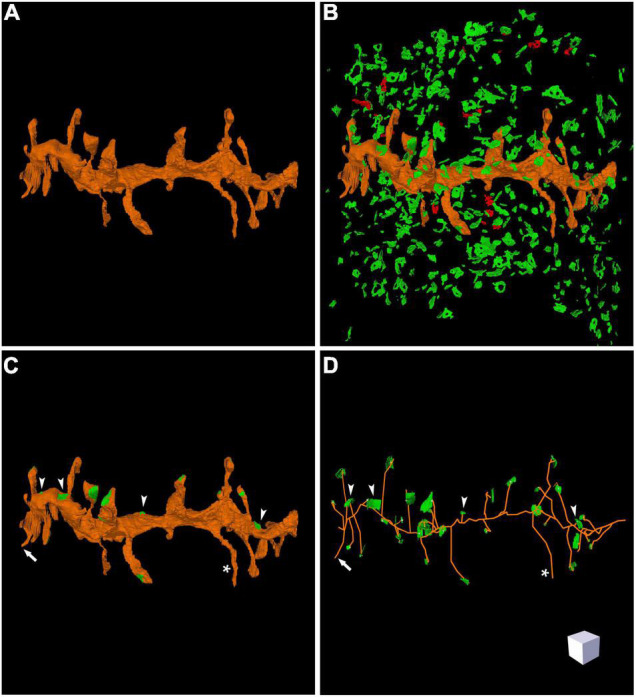
Three-dimensional reconstruction of a labeled dendritic segment and the surrounding synaptic junctions. **(A)** Reconstructed dendritic segment showing multiple dendritic spines. **(B)** The synaptic junctions present in the neighboring tissue have been segmented and reconstructed. Excitatory synaptic junctions are represented in green and inhibitory synaptic junctions are shown in red. **(C)** The same dendritic segment has been rendered with its corresponding synaptic junctions (green). Most synapses are established on dendritic spines, but synapses on the dendritic shaft are also visible (arrow heads). One of the dendritic spines does not establish any synaptic contact (asterisk). **(D)** Skeletonized representation of the same dendrite with its corresponding synapses. Arrow heads point to synaptic junctions that are established on the dendritic shaft; the asterisk indicates one non-synaptic dendritic spine; the arrow points to a dendritic spine that was truncated by the edges of the stack of serial images, so no synapse could be found. Scale cube is 1 μm on each side. See also Supplementary Video 2.

**FIGURE 5:**
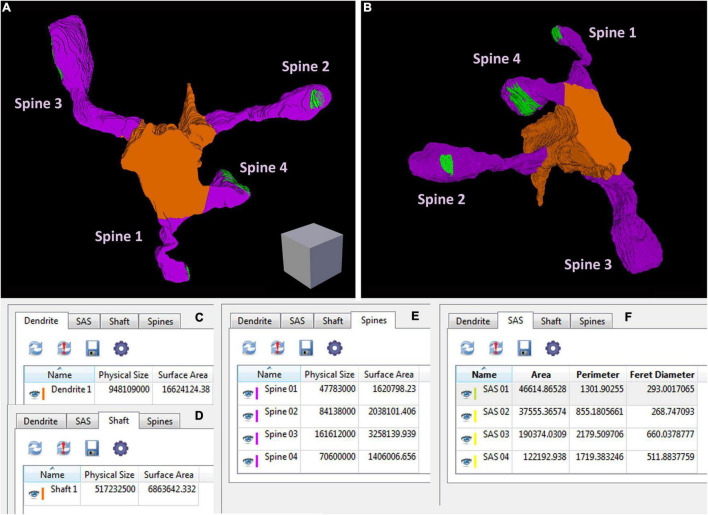
An example of geometric information obtained from a reconstructed dendritic segment and its corresponding dendritic spines and synaptic contacts (see also Supplementary Video 3). **(A,B)** A dendritic segment has been reconstructed with Espina software. Four dendritic spines (purple) arise from the dendritic shaft (orange). The synaptic junctions have been represented in green. Only complete dendritic spines have been represented in this example. In Supplementary Video 3 we have also represented dendritic spines that have been truncated by the edges of the stack. Once the structures have been reconstructed, the software provides different geometric parameters. **(C)** Espina tab for the total volume of the dendritic segment, indicated as “Physical size” and expressed in cubic nm. In this particular example, the estimation of the total volume comprises the dendritic shaft and all dendritic spines reconstructed as a whole, including spines that have not been represented in this figure but are shown in Supplementary Video 3. The surface area of the dendrite is expressed in square nm. **(D)** This tab shows the volume and surface area of the dendritic shaft alone. **(E)** The volume and surface area of each dendritic spine, reconstructed individually. **(F)** The area, perimeter and Feret diameter of the synaptic apposition surface, have been calculated for the four synaptic contacts. These and other parameters can be exported by Espina for further analysis (Espina software can be downloaded from https://cajalbbp.es/espina/). Scale cube is 0.5 μm on each side.

Espina software (or any other dedicated 3D software) can also be used for the segmentation and 3D reconstruction of the structures of interest. In this example, we have reconstructed the labeled dendritic segment, the synaptic junctions that it establishes, and the synapses located in the surrounding tissue (Figure 4). The labeled dendritic segment can also be skeletonized, providing a simplified version of the structure with its corresponding connections (Figure 4D). Segmentation is performed by shading the object of interest in the serial sections. Once segmented, any object can be rendered in three dimensions, visualized and measured by the software (Figure 5 and Supplementary Video 3). Quantitative information can be obtained from 3D segmentations, including the density and size of synaptic junctions, and the length, surface, and volume of the dendrite. For example, a dendritic segment can be reconstructed with its corresponding dendritic spines and synaptic contacts (Figure 5 and Supplementary Video 3). In this example, volumes and surfaces of the dendrite and its dendritic spines have been obtained with Espina software. In the case of synapses, the Feret diameter and synaptic apposition surface have been calculated (Morales et al., 2013).

## Discussion

The main advantage of using fixed tissue for intracellular injections is that you can select the region of interest very precisely. Using a fluorescent nuclear stain, it is possible to not only choose a particular brain area or layer, but also select the particular cell type to be injected. Neuronal nuclei are usually large and round, so neuronal somata are the easiest to locate. Injections are not limited to neurons; other cell types such as astrocytes can also be injected, on the basis of their smaller, irregularly shaped nuclei (Kikuchi et al., 2020). The main limitation of the injection of single cells in fixed sections is that only local processes can be studied, and some processes, especially in large neurons, will be truncated since they usually span farther than the section thickness. However, multiple injections can be performed in the same section, and different planes of section can be used to focus on different cell compartments. For example, coronal or sagittal sections are suitable for the study of the apical dendrites of cortical pyramidal cells, while sections that are parallel to the cortical surface are best suited for the study of basal dendrites (e.g., see Benavides-Piccione et al., 2006). Thus, even though some of the processes of single neurons may be truncated, it is possible to design a sampling strategy that provides a general view of the target cell type using multiple injections and different sectioning orientations.

Re-sectioning the original 250 μm-thick vibratome section into a series of thinner sections (7 μm thick) increases the precision by which we can select and image any compartment of the target cell. We selected a thickness of 7 μm to optimize the field of view and resolution of serial sections obtained by FIB-SEM. We routinely use a field of view of 10.24 μm × 7.68 μm, with a resolution, or voxel size, of 5 nm × 5 nm × 20 nm. With these settings, acquisition time per frame is between 2 and 4 min, using frame integration as the noise reduction method. Other settings can of course be used, but they must be carefully selected as a compromise solution between the desired resolution, field of view and acquisition time. For example, if we increase the field of view, the acquisition time per frame will also increase, so the number of serial images per session will decrease. Similar re-sectioning protocols have been described (Hayworth et al., 2015; Kislinger et al., 2020). The main advantages of our method are its simplicity and the fact that it does not require specialized equipment or techniques for thick sectioning and collection of sections.

Selecting the appropriate resolution is especially important given that the freshly milled surface is imaged and immediately milled again to acquire the next image, meaning that the series of images can only be obtained once. This is often viewed as a disadvantage (Titze and Genoud, 2016; Schifferer et al., 2021), although —in practice— this is not the case if an adequate sampling strategy is carefully planned. For example, the dendritic segment shown in Figures 2–4 is around 12 μm long. Even though it cannot be imaged again since it was milled by the FIB when it was sequentially imaged, we have many other similar segments in the same cell that we could also use. In fact, we routinely use a multiple sampling approach so that we obtain multiple stacks of images from the same region from different individuals. In this way, the information we finally obtain from any given brain area, layer or cell comes from multiple samples that can be statistically analyzed. Although our method has been designed for the study of targeted, relatively small regions of identified cells in great detail, it is compatible with other serial section methods that allow a wider field of view. These methods include serial block-face SEM (Denk and Horstmann, 2004; Briggman and Denk, 2006), ATUM (Schalek et al., 2011; Hayworth et al., 2014) or multi-beam techniques (Eberle and Zeidler, 2018). However, these techniques have a lower resolution in the *Z*-axis, since section thicknesses of 20 nm or less, that are easily obtained by FIB milling, are not possible by mechanical sectioning. Recent developments in ion milling —such as gas cluster ion milling (Hayworth et al., 2020)— will offer wide fields of view with no loss of resolution in the *Z*-axis.

In our method, LY is only used to monitor the injection of HRP. Given that LY is visible under the fluorescence microscope, we use it to determine the position of the electrode tip, to ascertain that we have impaled the appropriate cell, to check if there is any leakage during the injection, and to help decide when the filling of a given cell has been completed. Once the cells have been filled, we proceed directly to the development of HRP, so LY no longer participates in the labeling process. We also tested the injection of LY alone, with subsequent photoconversion of DAB (e.g., see Lübke, 1993; Ohm and Diekmann, 1994; Hanani, 2012). However, we found that HRP consistently yielded a denser precipitate, less background staining and a more consistent filling of distal cell processes of neurons. The dense precipitate of DAB facilitates the identification and reconstruction of large structures such as dendritic spines, dendritic shafts or mitochondria. However, it masks smaller organelles such as synaptic vesicles, which may hinder the identification of presynaptic terminals of labeled cells. This limitation must be taken into consideration when designing any study and, if the analysis of axonal boutons or terminals is intended, it may be necessary to consider other methods of intracellular labeling, either in combination with the present technique or as an alternative to it.

Focused ion beam milling and scanning electron microscopy serial sectioning can be combined with several labeling methods. These include genetic labeling (Bosch et al., 2015, 2016) tract-tracing (Rodriguez-Moreno et al., 2018, 2020) and immunocytochemistry (Turégano-López et al., 2021). These methods allow the researcher to select a group of cells based on their origin, projections, or neurochemical characteristics. Intracellular injection of single cells adds the capability to select single neurons based on their location, with a precision of microns, both in the *X*-*Y* plane and the *Z*-axis. The morphological features of the injected cells can be analyzed at the light microscopic level, and multiple cells can be selected and studied in 3D at the electron microscopic level, as can multiple regions of the same cell. This provides a powerful tool to bridge the mesoscopic and nanoscopic scales of the brain and thus will help to establish the relationships between long-range projections and connectivity at the synaptic level. Finally, our method not only facilitates the mapping of the synaptic connectivity of single-labeled neurons, but also the analysis of the surrounding neuropil. Since the labeled processes can be located in different layers or subregions, this method can also be used to obtain data on the similar or different local synaptic organization that may exist at different portions of the labeled neurons.

## Data Availability Statement

The original contributions presented in the study are included in the article/Supplementary Material, further inquiries can be directed to the corresponding author.

## Ethics Statement

All procedures were approved by the Local Ethics Committee of the Spanish National Research Council (CSIC).

## Author Contributions

JD and AM-P designed the research. MT-L, AS, AK, and J-RR performed the research. MT-L, AS, and AM-P analyzed the data. MT-L, AS, AM-P, and JD wrote the manuscript. All authors contributed to the article and approved the submitted version.

## Conflict of Interest

The authors declare that the research was conducted in the absence of any commercial or financial relationships that could be construed as a potential conflict of interest.

## Publisher’s Note

All claims expressed in this article are solely those of the authors and do not necessarily represent those of their affiliated organizations, or those of the publisher, the editors and the reviewers. Any product that may be evaluated in this article, or claim that may be made by its manufacturer, is not guaranteed or endorsed by the publisher.

## Footnotes

1
https://imagej.net/software/fiji/
